# Alterations of oral microbiota and cytokines profile in children and young adults with type 1 diabetes

**DOI:** 10.3389/fendo.2025.1629185

**Published:** 2025-10-01

**Authors:** Luana Aldegheri, Eulalia Catamo, Nunzia Zanotta, Giuseppina Campisciano, Carolina Cason, Andrea Conti, Chiara Cipullo, Milena Cadenaro, Gianluca Tornese, Chiara Navarra, Manola Comar, Antonietta Robino

**Affiliations:** ^1^ Institute for Maternal and Child Health – IRCCS Burlo Garofolo, Trieste, Italy; ^2^ Department of Medicine, Surgery and Health Sciences, University of Trieste, Trieste, Italy

**Keywords:** type 1 diabetes, oral microbiota, cytokines, dysbiosis, 16S targeted sequencing

## Abstract

**Introduction:**

Type 1 diabetes (T1D) is a chronic autoimmune disease caused by the destruction of pancreatic β cells. Although T1D is characterized by an increased susceptibility to oral inflammatory diseases, studies investigating the relationship between oral microbiota, oral inflammation, and T1D remain limited and often report conflicting results.

**Methods:**

In this work, we compared both oral microbiota composition and cytokines profile of 75 children and young adults with T1D and 79 healthy controls (HC), using 16S rRNA gene targeted sequencing and magnetic bead-based multiplex immunoassays.

**Results:**

We found significant changes in both alpha and beta diversity, with a decrease in species richness (p-value<0.001) and an increase in species evenness (p-value<0.001). At the genus level, T1D participants exhibited a lower abundance of *Fusobacterium* (p-value<0.001) and a higher abundance of *Atopobium* and *Prevotella* (p-value=0.011 and p-value=0.022, respectively) compared to HC. Moreover, cytokines profile analysis showed lower levels of IFN-γ (p-value<0.001) and increased levels of IL-1β (p-value=0.01) in T1D compared to HC. An association between IL-1β levels and the abundance of *Prevotella* also emerged (p-value=0.001).

**Discussion:**

Overall, our findings suggest that in subjects with T1D changes in the oral microbiota composition may contribute to modify the immune response toward alteration of the cytokine profile.

## 1 Introduction

The oral cavity acts as the primary gateway for a multitude of pathogens, and it represents the second-largest microbial reservoir in the human body. Studies on the composition of the oral microbiome have revealed a highly diverse community of over 700 bacterial species, which are grouped into seven major phyla: Actinomycetota, Bacteroidota, Bacillota, Fusobacteriota, Pseudomonadota, Saccharibacteria, and Spirochaetota (1).

The balance and the composition of the oral microbiota are considered important in maintaining a healthy oral environment but also influencing overall well-being and preventing systemic diseases. Indeed, oral dysbiosis has been identified as a potential contributing factor in the development of various oral conditions, including dental caries and periodontal disease (2, 3). Moreover, emerging research indicates that the oral microbiome may serve as a reservoir for systemic infections and inflammation, with certain bacterial species linked to immune modulation and an increased risk of body-wide diseases, including autoimmune conditions.

For instance, type 1 diabetes (T1D), caused by the autoimmune response against pancreatic β cells, is one of the diseases characterized by oral microbiome alterations (4). Onset of T1D can occur at any age, but it often manifests itself in children, adolescents and young adults. In T1D subjects, impaired salivary gland function often leads to changes in saliva composition, including reduced salivary flow rate (dry-mouth symptom), acidification of the oral cavity, and subsequent shifts in the oral microbiome composition (4–6).

In particular, studies have observed that T1D subjects had lower microbial diversity in comparison to healthy individuals, along with an increased abundance of genera harboring opportunistic pathogens (7, 8). Moreover, an increasing body of evidence reports that alterations of the oral microbiome may affect gut microbial communities, indicating the presence of a complex bidirectional interaction between the two sites and suggesting that oral-cavity-driven gut microbiome changes may contribute towards the inflammatory processes involved in T1D (4).

This condition is, in fact, characterized by elevated levels of circulating inflammatory markers, and several studies have also reported the presence of increased levels of pro-inflammatory mediators in the gingival tissues of diabetic subjects, such as IL-1β (interleukin 1 beta), tumor necrosis factor (TNF-α), IL-6 (interleukin 6), IL-8 (interleukin 8), and macrophage inflammatory proteins (MIP-1α, MCP-1). On the other hand, decreased levels of anti-inflammatory markers were also detected in T1D subjects (9, 10). Taken together, these factors explain a deficiency in the protective mechanisms of the oral cavity in T1D subjects, which contributes to an increased susceptibility to inflammatory diseases.

Despite this evidence, to date, limited research with frequently contradicting findings has been conducted to describe the possible relationship between oral microbial composition and T1D.

Also, the interplay between oral microbiome and inflammatory markers in T1D remains poorly understood, since that most previous studies have investigated oral microbiota and inflammatory markers separately, without providing an integrated view of host–microbiota interactions. In this light, the present work aims to simultaneously explore the oral microbiota composition and a broad panel of salivary cytokines in a cohort of children and young adults with T1D and healthy controls, helping to identify potential combined signatures that may contribute to T1D.

## 2 Materials and methods

### 2.1 Participants

In this study, 75 T1D subjects and 79 healthy controls (HC) were recruited from the Diabetes Unit and the Emergency Department of IRCCS Burlo Garofolo (Trieste, Italy). Inclusion criteria for T1D participants were: diagnosis of T1D for at least 1 year, age between 6 and 21 years, and absence of other types of diabetes mellitus (i.e., type 2, monogenic diabetes, cystic fibrosis-related diabetes). For HC enrolment, inclusion criteria were: age between 6 and 21 years, no diagnosis of T1D or any other diabetes forms, absence of obesity or other metabolic disorders, HbA1c ≤6% (≤42 mmol/mol), and no family history of diabetes. Additionally, for both groups, participants were excluded if they had taken antibiotics or anti-inflammatory drugs within 14-days prior to sampling.

The research project was approved by ethic committee (CEUR-2018-Em-323-Burlo). Before the enrollment all participants or their parents/guardians (for participants aged <18 years) provided written informed consent.

### 2.2 Demographic and clinical data

For all participants, demographic characteristics, including age, gender, and anthropometric measurements, were collected at the time of enrollment. Standard deviation scores of body mass index (BMI SDS) were calculated according to WHO reference charts using the Growth Calculator 4 software (http://www.weboriented.it/gh4/). HbA1c was measured with finger pricks using portable instrumentation at outpatient clinics (QuikRead go, A. De Mori S.p.A, Milan, Italy).

At the time of enrollment, a questionnaire on oral hygiene habits was administered to each participant. The survey, based on a scoping review of the literature, included questions on the frequency of daily oral hygiene, oral hygiene behavior, dental check-ups, and hours of fasting prior to the visit.

### 2.3 Saliva collection and DNA extraction

For each participant, 2 mL of saliva were collected at the time of enrolment. Subjects were required to avoid brushing their teeth with fluoride toothpaste for 12 hours prior to sample collection to avoid its antibacterial activity (11).

All saliva samples were immediately stored at −80 °C until further processing.

A total of 300 μL of each sample was used for DNA extraction, with a final elution volume of 50 μL. This was conducted using the Maxwell CSC instrument (Promega Srl, Madison, WI, USA) in accordance with the manufacturer’s instructions. Subsequently, the concentration (ng/µL) of the nucleic acid was determined using the NanoDrop^®^ 1000 spectrophotometer (Thermo Fisher Scientific, Waltham, MA, USA). The absorbance ratios at 260/280 and 260/230 were evaluated as indicators of the DNA purity.

### 2.4 16S targeted sequencing

The bacterial composition of the samples was assessed through 16S rRNA gene targeted sequencing. Library preparation was performed following the Illumina 16S Metagenomic Sequencing Library Preparation protocol (Illumina Inc., San Diego, California, CA, USA). The hypervariable V3-V4 region (~460 bp) of the 16S rRNA gene was amplified using primers 341F (5’-CCTACGGGNBGCASCAG-3’) and 805R (5’-GACTACNVGGGTATCTAATCC-3’), including Illumina overhang adapters sequences.

Amplicons were purified using AMPure XP beads (Beckman Coulter, Brea, CA, USA), indexed with the Nextera XT Index Kit (Illumina, San Diego, CA, USA), and quantified using a Qubit^®^ 2.0 Fluorimeter (Invitrogen, Carlsbad, CA, USA) in combination with the Qubit^®^ dsDNA HS Assay Kit (Thermo Fisher Scientific, Waltham, MA, USA).

Finally, an equal amount of each amplified product was mixed into a single batch to generate the pooled library, according to the manufacturer’s instructions.

In preparation for cluster generation and sequencing, the pooled library was denaturated with 0.2 N NaOH, diluted with hybridization buffer, and then heated denatured before 300 paired-end sequencing on an Illumina MiSeq platform (Illumina, San Diego, CA, USA). A no template control reaction was also added using Rnase/Dnase-free distilled water, moreover the final library mixture contained 15% PhiX to serve as an internal control.

Raw sequence data were processed using QIIME 2 software v2024.5 (https://qiime2.org). Denoising and chimera removal were performed with DADA2 v2022.2.0, and high-quality reads (Q ≥ 30) were retained. The negative control did not yield any high-quality sequences after DADA2 filtering, indicating the absence of detectable contaminant DNA in our dataset.

Taxonomic classification was carried out in QIIME 2 by comparing representative sequences to the SILVA v138.2 reference database. To handle variability in sequencing read depth, bacterial abundance data were normalized using Total Sum Scaling (TSS).

### 2.5 Cytokines analysis

In all participants, a panel of 27 cytokines, chemokines and growth factors was assessed in saliva using magnetic bead-based multiplex immunoassays (Bio-Plex Pro™ human cytokine 27-plex panel; Bio-Rad Laboratories, Milan, Italy), according to the manufacturer’s instructions.

The concentrations of the immune soluble factors were determined using the Bio-Plex-200 system (Bio-Rad Corp., Hercules, CA, United States) and Bio-Plex Manager software (v.6; Bio-Rad). Results were reported as median fluorescence intensity (MFI) and concentration (pg/mL).

### 2.6 Data processing and statistical analysis

Sample characteristics were summarized using contingency table. Categorical variables were presented as percentages (%), while continuous variables were assessed for distributional normality (via skewness and kurtosis) and reported as mean ± standard deviation (SD).

Differences between HC and T1D subjects were evaluated using the χ^2^ test or Fisher’s test for categorical variables, and the t test for continuous variables.

For statistical analyses, bacterial abundance and cytokines concentration were log-transformed.

All log-transformed bacterial relative abundance values inferior to the relative abundance value at 2 left standard deviations from the mean value of log-transformed bacterial relative abundance of each subject were set to 0.

For microbiota analysis, alpha diversity (microbiome diversity within a community) was assessed using Chao1, Shannon, Simpson and Pielou indices by linear regression analysis. These indices were calculated to evaluate the richness and diversity at the community level. Specifically, the Chao1 index estimates microbial richness; the Shannon index considers both the richness and evenness of species; the Simpson index is a diversity index that quantifies the biodiversity in a given habitat; and the Pielou index measures the degree of evenness of microbial distribution.

The Bray–Curtis dissimilarity index was computed in MicrobiomeAnalyst (12) to assess the beta diversity, which measures the similarity or dissimilarity of the analyzed groups, visualized through the principal coordinate analysis (PCoA), and compared by the PERMANOVA test.

To highlight the differences in the microbial composition between groups, we applied the LEfSe test and results were adjusted for multiple testing using the False Discovery Rate (FDR) method. Prior to these tests, taxa with prevalence in samples <20%, relative abundance <1%, and singletons were removed.

Moreover, linear regression analysis was also performed to confirm results obtained by LEfSe analysis.

To explore differences in cytokines profile among T1D and HC individuals a combination of PCoA and Volcano Plot were employed. PCoA was computed using vegan, ape and ggplot2 R packages, while Volcano Plot using EnhancedVolcano R package with the following criteria: p-value<0.05 after FDR correction and log2 fold change>1. Moreover, as for microbiome data, linear regression was also performed to confirm significant results.

For regression analysis of both bacteria abundance and cytokines levels, the dependent variable was the log-transformed value, while the independent variable was the disease status (T1D vs. HC). These regressions were performed by adjusting data for gender, age, fasting hours and oral hygiene frequency.

Additionally, in the whole sample, to assess the association between cytokine levels (dependent variable) and bacterial genera (independent variable) linear regression analysis was also applied, including gender, age, fasting hours, oral hygiene frequency and disease status as covariates.

In the regression models, covariates were selected based on their biological plausibility and potential role as confounding factors.

All statistical analyses were performed with R software (V4.2.2., www.r-project.org, accessed on 31 October 2022).

## 3 Results

### 3.1 Population

The study included 75 participants with T1D (49% males) with a mean age (± SD) of 15.2 ± 3.9 years, and a control group of 79 healthy participants (48% males) with a mean age (± SD) of 13.4 ± 4.5 years. No gender differences emerged between T1D and healthy participants, while age and BMI-SDS differed significantly among the two groups (p-value=0.008 and p-value=0.023, respectively). As expected, significant differences also emerged in HbA1c values (p-value<0.001).

As regard oral hygiene habits, 90% of T1D patients brush their teeth at least once a day compared to 100% of HC (p-value=0.021). No other significant differences have emerged (**Supplementary Table S1**).

### 3.2 Oral microbiota in T1D and healthy controls

**Figure 1:**
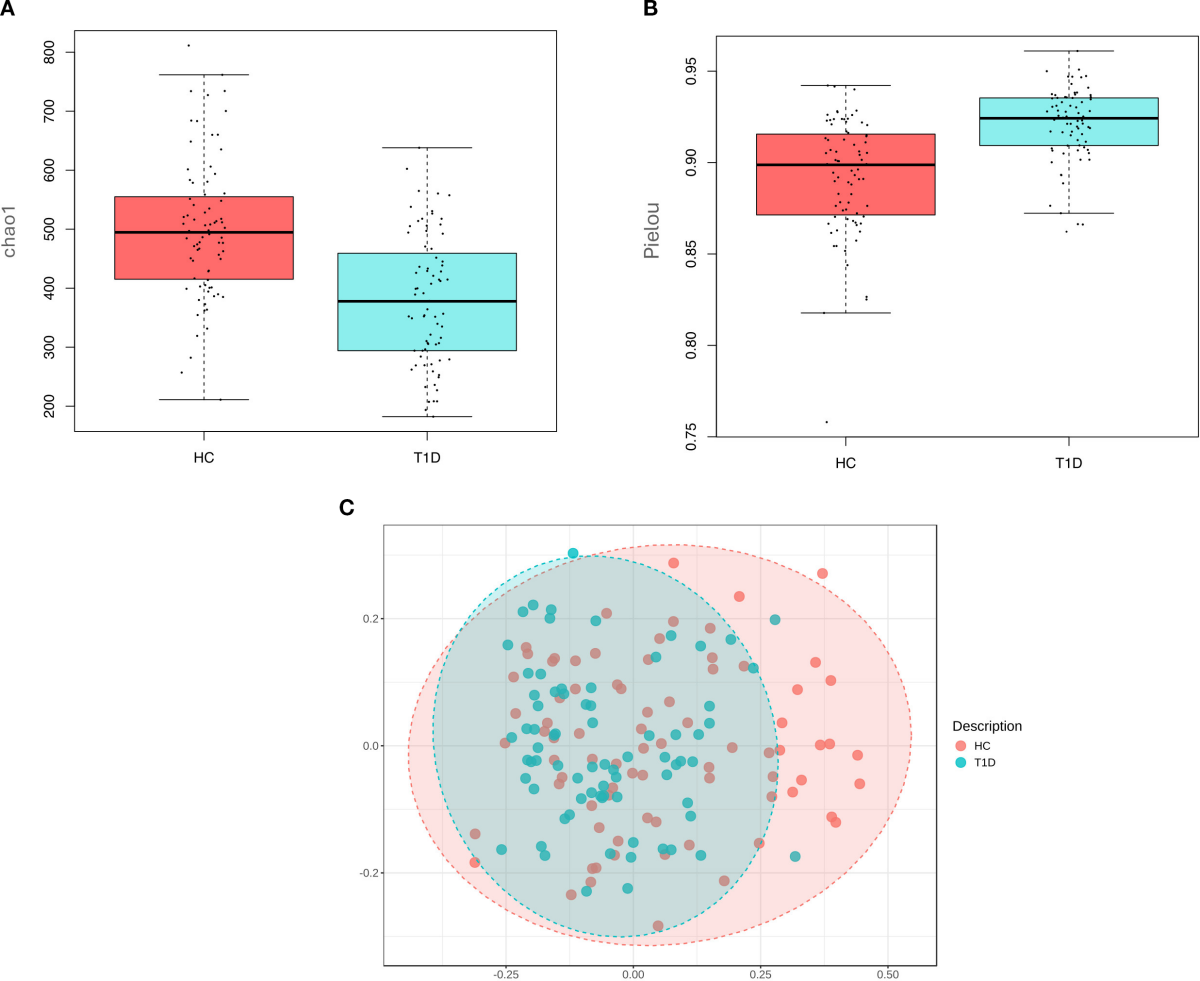
Microbiota characteristics in the saliva of healthy controls and type 1 diabetes subjects. Chao1 index **(A)** and Pielou index **(B)** significantly differ (p-value<0.001) in healthy controls (HC) and type 1 diabetes subjects (T1D). Principal coordinate analysis (PCoA), computed in MicrobiomeAnalyst, shows a significant separation between the two groups (p-value=0.001) **(C)**.

Comparing the oral microbiota between T1D and HC subjects, we observed a significant reduction in alpha diversity in the T1D group. Specifically, regression analysis (with gender, age, fasting hours, oral hygiene frequency as covariates) showed that species richness, as assessed by Chao1 index, was significantly lower in T1D subjects compared to HC (p-value<0.001, beta=-94.4) (**Figure 1A**). Conversely, species evenness, measured by Pielou index, was higher in T1D subjects (p-value<0.001, beta=0.024) (**Figure 1B**). Shannon and Simpson indices did not differ among the two groups.

Beta diversity, based on Bray-Curtis dissimilarity, also revealed a significant separation of T1D subjects from HC (p-value=0.001) (**Figure 1C**), meaning a different oral microbiota composition among the two groups.

**Figure 2:**
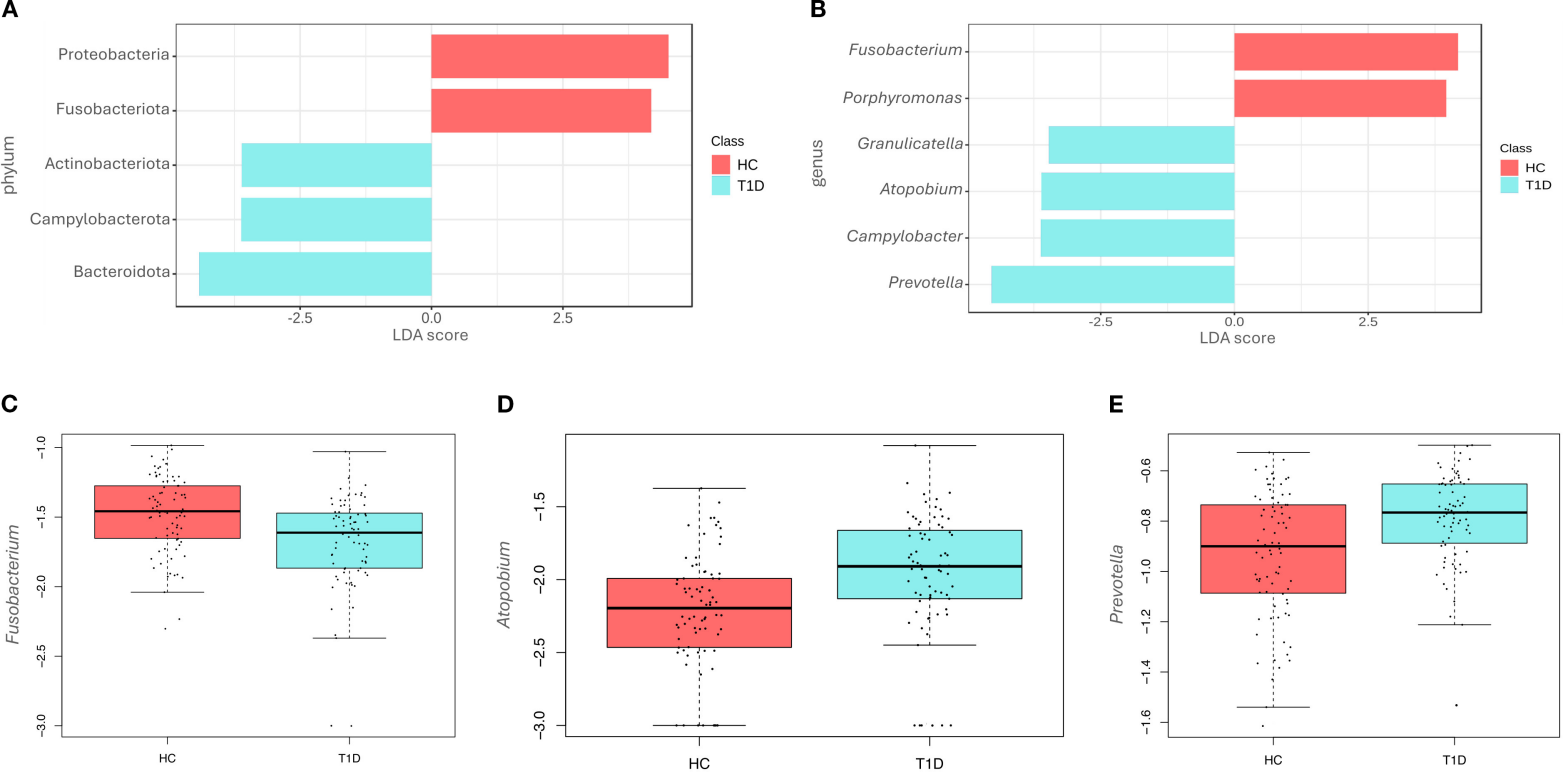
Differences at phyla and genera level among healthy controls and type 1 diabetes subjects. LEfSe analysis shows the differentially abundant phyla **(A)** and genera **(B)** in the saliva of healthy controls (HC) and type 1 diabetes (T1D) subjects. The Linear Discriminant Analysis (LDA score) indicates the effect size of each differentially represented taxon, with higher scores reflecting greater differences in relative abundance. Regression analysis shows differences at genus level between the two groups for *Fusobacterium* (p-value<0.001) **(C)**, *Atopobium* (p-value=0.011) **(D)** and *Prevotella* (p-value=0.022) **(E)**.

To better understand changes in microbiota composition at the phylum and genus levels between T1D and HC, LEfSe analysis was conducted. At the phylum level, significant differences were observed in the relative abundance of Proteobacteria, Fusobacteriota, Actinobacteriota, Campylobacterota, and Bacteroidota, based on effect size (LDA score). In particular, Actinobacteriota (p-value=0.004), Campylobacterota (p-value<0.001), and Bacteroidota (p-value=0.002) were enriched in T1D, while Proteobacteria (p-value=0.047) and Fusobacteriota (p-value<0.001) were more abundant in HC (**Figure 2A**).

Differences were also observed at the genus level (**Figure 2B**), with the T1D microbiota characterized by significantly higher levels of *Granulicatella* (p-value=0.012), *Atopobium* (p-value=0.004), *Campylobacter* (p-value<0.001), and *Prevotella* (p-value<0.001). In contrast, higher levels of *Fusobacterium* (p-value<0.001) and *Porphyromonas* (p-value=0.012) were observed in the HC group. No statistically significant differences were detected at the species level.

Regression analyses were also performed to account for confounding factors as gender, age, fasting hours and hygiene frequency. Results of regression analysis confirmed only in part findings of LEfSe analysis. In fact, at the genus level, *Fusobacterium* was significantly reduced in T1D (p-value<0.001, beta=-0.13) (**Figure 2C**), while *Atopobium* (p-value= 0.011, beta=0.10) (**Figure 2D**) and *Prevotella* (p-value=0.022, beta=0.09) (**Figure 2E**) were increased. *Porphyromonas, Granulicatella*, and *Campylobacter* did not show any significant differences, contrary to the results of the LEfSe analysis.

### 3.3 Cytokine profile in T1D and healthy controls

**Figure 3:**
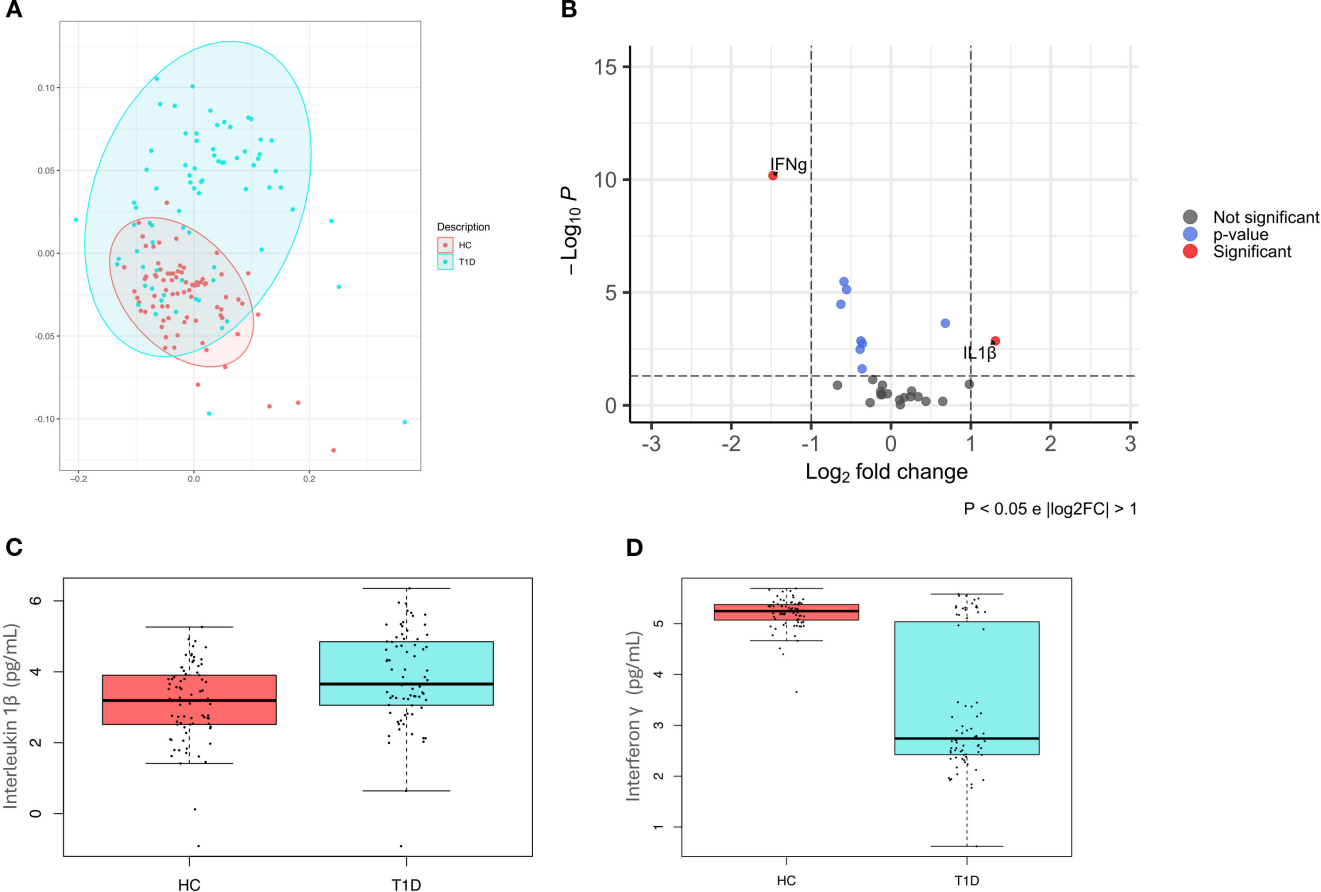
Cytokines pattern in healthy controls and type 1 diabetes subjects. **(A)** Principal coordinate analysis (PcoA) shows a significant difference (p-value=0.001) in healthy controls (HC) and type 1 diabetes subjects (T1D); **(B)** Volcano plot highlights discriminant oral cytokines in terms of their fold change>1 (*X* axis) and logarithm of Mann–Whitney *U* test p-value<0.05 (*Y* axis). **(C)** Boxplot comparing significant differences in IL-1β (p-value=0.01) **(C)** and IFN-γ (p-value<0.001) **(D)** levels, expressed in pg/mL.

Analysis of a panel of 27 cytokines, including chemokines and growth factors, showed that T1D were significantly distinguishable from HC (p-value=0.001) (**Figure 3A**).

A volcano plot was then used to identify discriminant oral cytokines among the two groups (**Figure 3B**). Results indicated that IL-1β was increased in T1D compared to HC, while IFN-γ levels were higher in HC.

Regression analyses have confirmed significant differences among T1D and HC for both IL-1β (p-value=0.01, beta=0.22) (**Figure 3C**) and IFN-γ (p-value<0.001, beta=-0.80) (**Figure 3D**).

### 3.4 Association between oral bacteria species and oral cytokines

The relationship between bacterial genera and cytokines level was analyzed by regression analysis, using gender, age, fasting hours, hygiene frequency in addition to disease status as covariates. These analyses included only bacterial genera and cytokines previously identified as distinctive for T1D.

A significant direct association was found between IL-1β levels and the relative abundance of the *Prevotella* genus (p-value=0.001, beta=0.73), showing that higher *Prevotella* abundance is correlated with higher IL-1β levels (**Table 1**). No other associations have emerged.

**Table 1:** Association between oral bacterial genera and cytokine levels assessed by linear regression models.

Bacterial genus	Cytokine	Beta	P-value
*Fusobacterium*			
	IL-1β	0.07	0.654
	IFN-γ	-0.11	0.383
*Atopobium*			
	IL-1β	0.19	0.082
	IFN-γ	-0.02	0.811
*Prevotella*			
	IL-1β	0.73	**0.001**
	IFN-γ	-0.24	0.216

Age, gender, fasting hours, oral hygiene frequency and disease status were used as covariates. Statistically significant results (p-value<0.05) were reported in bold; beta indicated the estimated effect.

## 4 Discussion

In the present study, we identified alterations in the oral microbiota and oral cytokines profile of children and young adults with T1D, providing new insights into the interaction between oral microbial composition and inflammation in T1D.

Consistent with previous studies, we found significant differences between the oral microbiota of T1D subjects and those of HC (7, 13, 14). Specifically, our analysis revealed significant changes in both alpha and beta diversity, with a notable decrease in species richness and an increase in species evenness. This pattern suggests the presence of a decreased microbial diversity in T1D subjects. Therefore, these findings are consistent with previous research conducted in both human and animal models, which has demonstrated that diabetes is associated with reduced microbial diversity in various body sites. While the gut microbiota has been widely studied in the T1D context, our results suggest that similar dysbiotic condition may also occur in the oral microbiome, which remains relatively under-investigated (15–18).

Moreover, in our work, at the genus level, both LEfSe and regression analysis revealed lower abundance of *Fusobacterium* and higher abundance of *Atopobium* and *Prevotella* in T1D participants. In contrast, other taxa identified by LEfSe (e.g., *Porphyromonas*, *Granulicatella*, *Campylobacter*) were not confirmed by regression models with covariate adjustment, suggesting potential confounding effects.

*Fusobacterium* is a common anaerobe in the oral microbiota of healthy individuals, essential in oral biofilm formation across the buccal cavity, tongue, and subgingival plaque. In fact, it is known for its role in preserving the overall architecture and ecological balance of the oral microbiome (19). The reduced abundance of *Fusobacterium* observed in T1D subjects, in agreement with prior findings (7), suggests that its depletion may contribute to the development of oral dysbiosis in T1D.

Conversely, both *Prevotella* and *Atopobium* were enriched in T1D group. These genera have been previously associated with systemic diseases, including diabetes, characterized by an increase susceptibility to periodontitis (20).

*Atopobium*, a genus commonly found in the oral cavity, has been linked to anaerobic environments and inflammatory conditions (20); to our knowledge, no prior studies have directly associated it with T1D. Most probably, the increased abundance of *Atopobium* may indicate an altered microbial community contributing in T1D subjects to an inflammatory oral environment.

*Prevotella* is a commensal genus known for its acetate production and early colonization of oral mucosa and dental plaques. It has been implicated in the promotion of oral inflammation and has frequently been observed in individuals with metabolic disorders, including diabetes (21). In a prior study a decreased relative abundance of *Prevotella* in new-onset T1D subjects in the acute phase have been already reported (17), while only a decreasing trend have emerged in T1D participants in the chronic phase, as found in our study including only T1D participants with at least 1 year of disease duration. Our findings suggest that in the context of T1D, the elevated levels of *Prevotella* may contribute to an enhanced inflammatory response, exacerbating oral tissue damage and increasing susceptibility to oral diseases (22). Moreover, the rise in *Prevotella* in T1D may also reflect adaptation to the altered immune environment within the oral cavity of T1D patients. Metabolic interactions between *Prevotella* and other anaerobes, such as *Atopobium*, have been suggested, with *Prevotella* producing short-chain fatty acids (SCFAs) and succinate that can be utilized by cohabiting species within oral biofilms (23). Accordingly, an increase in *Prevotella* abundance may create favorable conditions that promote the growth of *Atopobium*.

Supporting this fact, in the present work cytokines profile was also analyzed, showing an overall difference between T1D and HC. Specifically, T1D subjects exhibited lower levels of IFN-γ, a pro-inflammatory cytokine that play a complex and dual role in both protection and destruction of β-cells (24). Our results are supported by other studies on both T1D and type 2 diabetes, reporting that the capacity of T cells to produce IFN-γ is decreased in diabetic patients, and that the reduction of IFN-γ production makes β-cells highly susceptible to viral infection, which lead to diabetes mellitus development (25, 26). Cytokine analysis also showed increased levels of IL-1β in T1D compared to HC, in agreement with literature (27). Moreover, in the present study, an association between IL-1β levels and the abundance of *Prevotella* emerged.

IL-1β is a pro-inflammatory cytokine of pancreatic β cells by promoting apoptosis (27). In fact, IL-1β is primarily produced by monocytes and macrophages and is involved in both dysfunction and destruction increased in subjects with newly diagnosed T1D, where it likely acts as an early inflammatory signal in the disease onset (28), but it is also found to be high in individuals with long-standing T1D (29).

Moreover, emerging studies have linked the genus *Prevotella* with various inflammatory conditions mediated by Th17-related immune responses, including periodontitis, bacterial vaginosis, rheumatoid arthritis, metabolic disorders. More specifically, *Prevotella* predominantly activates Toll-like receptor 2 (TLR2), leading to the production of Th17-related cytokines, including the pro-inflammatory IL-1β (22). This evidence supports our findings, suggesting that changes in the oral microbiota composition, such as an overrepresentation of *Prevotella*, can alter the immune response toward a pro-inflammatory profile characterized by elevated IL-1β levels. This, in turn, may contribute to the development or exacerbation of chronic inflammatory diseases.

Interestingly, targeting *Prevotella*-induced IL-1β signaling might also offer novel therapeutic opportunities in managing chronic inflammation in T1D. IL-1β inhibitors are becoming the standard of therapy for several conditions, and microbiota modulation could enhance treatment efficacy (30).

Although the present investigation has allowed the detection of changes in both oral microbiota composition and inflammatory profile in T1D, it is subject to some limitations. For example, dental health status and the presence of oral diseases were not evaluated and additional data that may influence oral microbiota (i.e., duration of diabetes, glycemic control, diet) were not assessed.

In conclusion, our study has provided a deeper insight into the importance of the oral microbiome in modulating oral inflammation in T1D, suggesting the need for early and regular dental care in T1D patients.

However, further studies are needed to better understand the underlying mechanism and the reciprocal interaction between oral microbiota composition, oral inflammation and T1D.

## Data Availability

The datasets presented in this study can be found in online repositories. The names of the repository/repositories and accession number(s) can be found below: https://www.ncbi.nlm.nih.gov/, PRJNA1262893.
