# Supplementary material for: Alterations of oral microbiota and cytokines profile in children and young adults with type 1 diabetes
Source: Front Endocrinol (Lausanne). 2025 Oct 1;16:1629185. doi: 10.3389/fendo.2025.1629185 (PMC12520906; doi:10.3389/fendo.2025.1629185)
Supplement: Supplementary file 1 [file Table1.docx]

**Table S1.** Sample characteristics and oral hygiene habits in healthy controls and type 1 diabetes subjects.

Data are shown as percentage (%) and mean and standard deviation (mean±SD).

Differences among healthy controls (HC) and type 1 diabetes (T1D) subjects were computed by $\chi$^2^ test or Fisher’s exact test for categorical variables, and by t test for continuous variables.

Significant p-values are shown in bold. Statistical significance was set at a p-value ≤0.05. BMI SDS = BMI standard deviation scores.

|  | **HC**  (n=79) | **T1D**  (n=75) | **p-value** |
| --- | --- | --- | --- |
| **Sample Characteristics** |  |  |  |
| Sex, male % | 48% | 49% | 1 |
| Age years, mean±SD | 13.4±4.5 | 15.2±3.9 | **0.008** |
| BMI SDS, mean±SD | -0.44±1.15 | 0.06±1.08 | **0.023** |
| HbA1c %, mean±SD | 5.26±0.34 | 7.52±1.24 | **<0.001** |
| **Oral Hygiene Habits** |  |  |  |
| Annual dental check-up, yes % | 94% | 93% | 1 |
| Daily toothbrushing, yes % | 100% | 90% | **0.021** |
| Toothbrushing frequency, yes % |  |  | 0.37 |
| 1/day | 4% | 0% |  |
| 2/day | 15% | 18% |  |
| 3/day | 81% | 82% |  |
| Toothbrushing type, yes % |  |  | 0.19 |
| Manual | 52% | 63% |  |
| Electric | 30% | 29% |  |
| Both | 18% | 8% |  |
| Fasting hours (h) prior to the visit |  |  | 0.24 |
| <2h, % | 49% | 62% |  |
| ≥2h, % | 51% | 38% |  |
